# Microfluidic Organ-on-a-Chip System for Disease Modeling and Drug Development

**DOI:** 10.3390/bios12060370

**Published:** 2022-05-27

**Authors:** Zening Li, Jianan Hui, Panhui Yang, Hongju Mao

**Affiliations:** 1 State Key Laboratory of Transducer Technology, Shanghai Institute of Microsystem and Information Technology, Chinese Academy of Sciences, Shanghai 200050, China; lizening211@mails.ucas.ac.cn (Z.L.); jiananhui2@mail.sim.ac.cn (J.H.); yph@mail.sim.ac.cn (P.Y.); 2 Center of Materials Science and Optoelectronics Engineering, University of Chinese Academy of Sciences, Beijing 100049, China

**Keywords:** organ-on-a-chip, microfluidics, drug development, disease modeling

## Abstract

An organ-on-a-chip is a device that combines micro-manufacturing and tissue engineering to replicate the critical physiological environment and functions of the human organs. Therefore, it can be used to predict drug responses and environmental effects on organs. Microfluidic technology can control micro-scale reagents with high precision. Hence, microfluidics have been widely applied in organ-on-chip systems to mimic specific organ or multiple organs in vivo. These models integrated with various sensors show great potential in simulating the human environment. In this review, we mainly introduce the typical structures and recent research achievements of several organ-on-a-chip platforms. We also discuss innovations in models applied to the fields of pharmacokinetics/pharmacodynamics, nano-medicine, continuous dynamic monitoring in disease modeling, and their further applications in other fields.

## 1. Introduction

Microfluidics are a method to precisely control and manipulate micro-scale fluids, referring especially to technology using sub-micron structures for biological and chemical experiments. It only needs dozens or hundreds of nanoliter scale fluidic samples or reagents to complete mixing, separation, transport, etc. [1]. With the advancement of micro- and nano-machining, microfluidic structures such as micro-valves and pumps can be fabricated. A micro total analysis system (μTAS) has furthermore been developed, also known as a lab-on-a-chip (LOC) [2]. The pressure-driven control valve [3] not only enhances the micro-scale fluidic control with high integration and accuracy, but also promotes the study of more complex interactions of various molecules and cell lines [4].

In the early 1990s, A. Manz et al. first prototyped the microfluidic chip with structures designed for capillary electrophoresis experiments [5]. From 2000 to 2005, microfluidics-related technology developed dramatically. In 2002, Todd Thorsen et al. published a paper in *Science* entitled “Large-scale Integration of Microfluidic Chips”, in which they designed large-scale complex flow control circuits based on microvalves [6]. This made people realize the potential usage of this structure in micro-scale fluidic control. Currently, microfluidics has been utilized in many emerging applications, such as digital microfluidics, organs-on-chips (OoCs), point-of-care-testing, and wearable microfluidic devices [7,8,9,10].

The application of microfluidic OoCs in pre-clinical stages of drug development has great potential. Traditionally, drug development is a time-consuming, high cost, and low approval rate process. Most new drugs will be eliminated due to adverse reactions in the clinic. Between 2013 and 2014, about four out of every 1000 people in the United States were reported to have visited the emergency room for adverse drug events [11]. Animal models currently do not accurately predict how drugs will react in humans [12]. The 2D culture of human cells in vitro, although it can replace animal models to a certain extent, still has disadvantages in simulating the real cell microenvironment and results in the loss of cell function. Compared to traditional 2D cell cultures, a microfluidic LOC system can provide a more physiologically relevant environment for tissues and cells [13]. By integrating cell culture and microfluidic controlling units, OoCs can simulate the microenvironment of organs and tissues in a microfluidic structure. Hence, they could realize some physiological functions in vitro [14].

**Figure 1:**
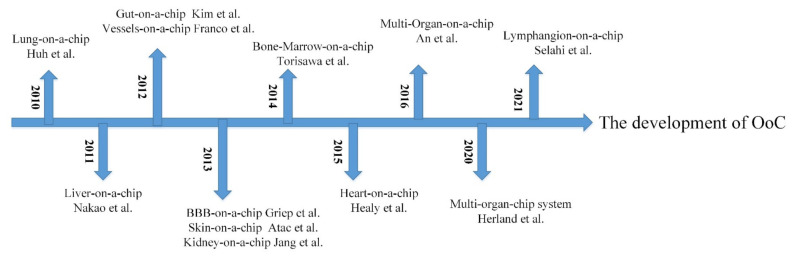
The development of OoC.

As the cornerstone for the development of OoCs, the cytosensor microphysiometer is a system for the bioassay, usually used to study cell function and measure biochemical parameters [15]. It is also used as a tool for toxicological and pharmacological testing in biosensors [16]. In the early 21st century, Mike Shuler first proposed the idea of using cells from different human organs to build human tissue on a chip to simulate the human environment [17]. The lung-on-a-chip fabricated by Huh et al. in 2010 attracted great attention [8]. In the following years, chips for liver [18], kidney [19], heart [20,21], gut [22], vessel [23], skin [24], bone marrow [25], and blood–brain barrier (BBB) [26] were also successfully constructed. Figure 1 shows the timeline of the OoC development. In this review, we mainly summarize the early models and recent research into various OoCs and introduce their applications in drug pharmacokinetics (PKs), pharmacodynamics (PDs) and nano-medicine, and disease modeling.

## 2. Design of an Organ-on-a-Chip

OoC is a cell culture device that simulates and realizes organ functional units in vitro. It uses a microfluidic device with a multi-layer structure to culture cells in microfluidics and simulates the microenvironment of tissues and organs in the human body. In addition, it can also add microsystems with mechanical activity to simulate the biological activities of organs [27]. Table 1 shows the human disease and clinical responses replicated in single organ chips.

The general principle of OoC design is the reductionist analysis of the target organ. First, it is necessary to understand the structure of target organs and simplify the physiological functions. Afterwards, four aspects need to be considered: cell selection and culture, control of dynamic flow-mediated perfusion, microstructure design of bionic organs, and mechanical movement of biological organs [28,29]. In this section, we will introduce the structure, application, and latest research results of several common OoCs.

### 2.1. Lung-on-a-Chip

**Figure 2:**
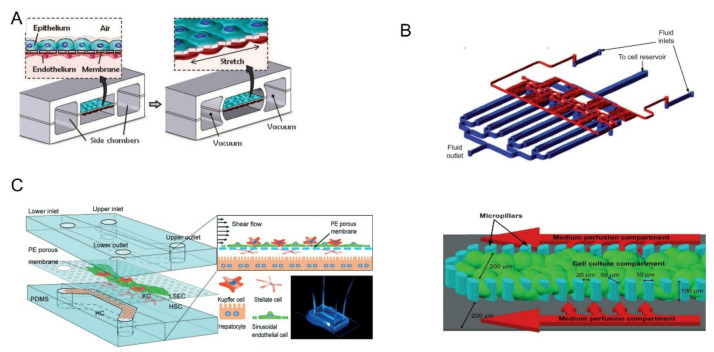
(**A**) The upper layer was alveolar epithelial cells and the lower layer was pulmonary microvascular endothelial cells. Biomechanical activity under respiration can be simulated by circulating vacuums on both sides of the chambers. Reprinted with permission from Ref. [8]. Copyright 2010 Science. (**B**) The multiplexed cell culture chip (blue) and the linear concentration gradient generator (red) were independently manufactured and connected with each other through stainless steel subcutaneous catheter. Single cell culture channels were divided by microcolumns into central cell culture compartments and two lateral culture medium infusion chambers. Reprinted with permission from Ref. [34]. Copyright 2009 Royal Society of Chemistry. (**C**) The upper and lower channels were made of PDMS and separated by a PE film. Four kinds of cells were distributed layer-by-layer on both sides of PE membrane. Reprinted with permission from Ref. [35]. Copyright 2017 Royal Society of Chemistry.

Before the fabrication of the lung-on-a-chip, it is necessary to understand the structure of the lung and its functional components. The alveolar-capillary unit of the lung consists of alveolar epithelial cells and pulmonary microvascular endothelial cells separated by a thin interstitium. Their cellular microenvironment is air and blood flow, respectively. Cyclic mechanical stretching occurs under the action of respiration [28]. Huh et al. used PDMS to produce a lung-on-a-chip model with a multi-layer microfluidic structure, as shown in Figure 2A [8]. The device was separated by a thin (10 μm) porous flexible membrane made of PDMS coated with ECM (fibronectin or collagen). Alveolar epithelial cells and pulmonary microvascular endothelial cells were cultured on either side of the membrane. By vacuuming and restoring the spaces on both sides, the breathing induced cyclic mechanical stretching could be simulated. In experiments exploring the toxic effects of silica nanoparticles, they found that respiratory exercise may significantly increase the pro-inflammatory activity of silica nanoparticles, which contributed significantly to the development of acute lung inflammation. The experimental results show that compared with the traditional static culture system, the multi-layer OoC can better reflect the motion state of tiny particles in the human body, providing a new means to study the principle of various diseases and drug toxicity.

Although the above-described lung-on-a-chip shows great potential in simulating organ function, there are still some problems. Firstly, the preparation of ultra-thin porous PDMS flexible membranes is technically challenging. In addition, PDMS can absorb small molecules such as drugs, which may affect the bioavailability of drugs when testing their effects [56]. Recently, Zamprogno et al. designed the second generation of lung-on-a-chip, whose biological membrane is made of proteins of the lung ECM, collagen, and elastin [32]. They experimentally tested that the soft collagen-elastin (CE) membrane was less adsorbed to rhodamine B compared to PDMS. They also found that CE film had good biodegradability, stretchability, and optical properties. The extracellular matrix (ECM) of the lung is critical for cell structural support, organ development, and the regulation of injury repair responses [57]. Therefore, the use of materials made from ECM molecules as biological membranes could be promising. Besides the vertical multi-layer structures, Zhang et al. also designed a lung-on-a-chip with a parallel structure. The device consists of three parallel channels for the infusion of alveolar epithelial cells, human vascular endothelial cells, and an extracellular matrix. The team first used the model to assess the lung toxicity of nanoparticles (ZnO and TiO_2_) [31]. They also used the same device to investigate how PM 2.5 would damage human lung function [58].

### 2.2. Liver-on-a-Chip

The liver is the largest internal organ of the human body, and it plays a key role in the synthesis and metabolism of various substances, especially in drug metabolism [59]. Therefore, it will be a new development direction to accurately test the metabolic process and toxicity of drugs in vitro. In 2009, Toh et al. designed a microfluidic liver cell chip for testing drug toxicity in vitro [34]. The device consisted of a multiplexed cell culture chip and a linear concentration gradient generator (the top half of Figure 2B). An array of micro-columns (the bottom half of Figure 2B) was arranged in the cell culture channel and the gap between the micro-columns was smaller than that of hepatocytes. Hence, the hepatocytes can be fixed in the central cell chamber. The team used the chip to test five hepatotoxic drugs, including acetaminophen and successfully obtained in vitro toxicity data related to the one in vivo. Hepatic sinusoids are an important part of the liver and play a vital role in the material exchange between liver cells and blood flow. Du et al. designed a multilayered liver-on-a-chip that replicated the in vivo configurations of liver sinusoidal endothelial cells (LSECs), Kupfer cells, hepatic stellate cells, and hepatocytes [35]. As shown in Figure 2C, a thin porous PE membrane was used to separate the cells. The platform was used for testing the effects of cell co-culture and fluid flow on protein secretion, cytotoxic metabolism, and immune response. The co-culture of LSECs with the other three types of liver cells were found to promote neutrophil recruitment in the bloodstream.

In addition to drug toxicity, the liver-on-a-chip also has the potential to study liver diseases. For example, nonalcoholic fatty liver disease (NAFLD), a condition in which fat is deposited in the liver for reasons other than alcohol consumption, is becoming one of the fastest-growing diseases in the world [60]. Many kinds of liver-on-a-chip have been used to study the pathology of hepatic steatosis [61]. For instance, Jeon et al. used their previously designed gut-liver chip to further explore the factors leading to hepatic steatosis and tested the anti-steatosis effect and mechanism of two drugs, turofexorate isopropyl (XL-335) and metformin [62]. They have simulated the absorption and accumulation of fatty acids in the gut and liver on the chip, demonstrating the absorption of fatty acids through the intestinal layer and subsequent deposition in liver cells [63,64]. The emergence of this multi-tissue model provides a new direction for understanding the metabolism of drugs in the human body and the pathology of various diseases.

### 2.3. Gut-on-a-Chip

The gut is an important organ for digestion and absorption. However, due to the complex microbial system in the gut, studies have been emerged to show that the pathogenesis of many diseases and the efficacy of drugs are closely related to it [65,66,67]. In 2012, Kim et al. created a gut-on-a-chip using the previously designed lung-on-a-chip structure [22,68]. The mechanical strains that mimic peristalsis and fluidic flow can accelerate the differentiation of intestinal epithelial cells, the formation of 3D villous structures, and the realization of intestinal barrier function. To test the effect of spatial structure on cell culture, Sung et al. created a microstructure that mimics human intestinal villi in hydrogels [69]. They found that the overall morphology of Caco-2 cell lines cultured on this structure was very similar to the human jejunal villi. Shim et al. fabricated a gut-on-a-chip based on a 3D villi structure [37]. The fluidic shear was considered and the two- and three-dimensional cell cultures were compared. These results showed that Caco-2 cells cultured under 3D villi had higher metabolic activity, but the shape of microvilli was poorly preserved.

**Figure 3:**
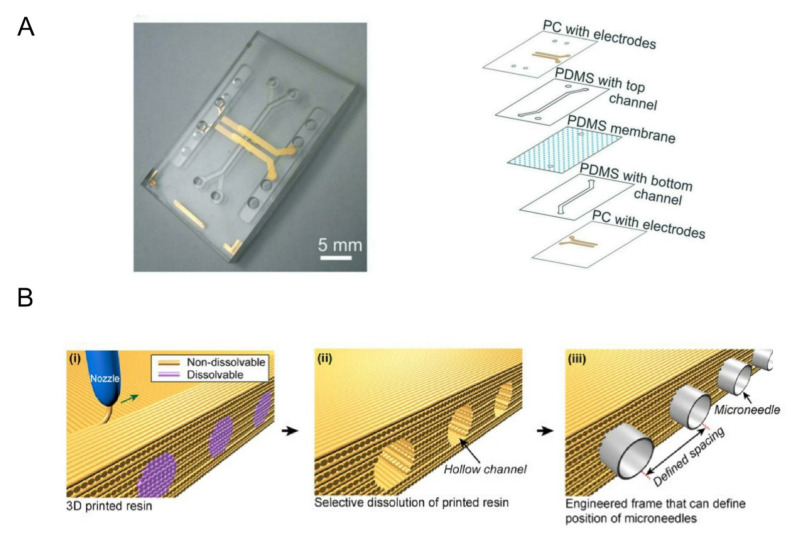
(**A**) Two PC substrates with integrated translucent gold electrodes were added to the upper and lower sides of the gut chip. Reprinted with permission from Ref. [72]. Copyright 2019 Royal Society of Chemistry. (**B**) Schematic illustration for fabrication of 3D printed frame. (**i**) Non-dissolvable and dissolvable resins were co-printed, and (**ii**) the dissolvable resin was selectively removed by submerging in DI water. (**iii**) Hollow channels were used for positioning microneedles. Reprinted with permission from Ref. [75]. Copyright 2015 American Institute of Physics.

Impedance spectroscopy is an important technique for measuring trans-endothelial or trans-epithelial resistance (TEER). By applying AC voltage within a certain frequency range, information such as resistance and cell layer capacitance can be obtained and cells can be detected [70,71]. In 2017, Henry et al. designed a TEER chip that can measure the barrier function and evaluate the viability of the organ chip in real time by combining the organ chip with the sensing electrodes [38]. In 2019, Helm et al. fabricated the gut-on-a-chip for a dynamic culture of human Caco-2 intestinal epithelial cells, as shown in Figure 3A [72]. The chip combined the impedance spectroscopy measurement with electrical stimulation, which can measure the function of the cell-layer barrier and detect the changes in intestinal intraepithelial villus differentiation non-invasively.

As we mentioned earlier, there are many micro-organisms in the gut that play an important role in the occurrence of some diseases and in metabolism. Jing et al. recently fabricated a novel peristaltic human intestinal microfluidic device to test intestinal injury and inflammatory response caused by *E. coli* [39]. The *E. coli* was first inoculated into the intestinal lumen for co-culture and the human macrophage U937 cells were then introduced. They found that the coverage and height of calyx and microvilli were affected, barrier function was impaired, and the secretion of inflammatory factors increased. Another experiment inoculated *L. casei* L5 BGB with *E. coli* in the intestinal lumen and found that these effects were significantly reduced, indicating that Lactobacillus casei and antibiotics can effectively reduce intestinal epithelial injury and inflammation. The system simulated various functions of the intestinal tract in the human body and has successfully been implemented in several microbial-related experiments. It will become an important platform for studying the role and mechanism of intestinal microorganisms in vitro.

### 2.4. BBB-on-a-Chip

The blood–brain barrier (BBB) is an important regulatory interface between blood and the central nervous system. It is important for maintaining the functions of the brain and central nervous homeostasis as it could prevent toxic substances from entering the brain. A variety of neurological diseases are associated with BBB injury [73,74]. Therefore, it is of great significance to establish an effective in vitro model for the further study of human brain health. In 2013, Griep et al. fabricated the BBB chip by cultured brain endothelial cells (hCMEC/D3) in microfluidic devices [26]. Integrated electrodes are used to measure TEER to analyze barrier tightness. Through experimental analysis, they found that the presence of shear stress enhanced endothelial barrier function, but TNF-α damage to barrier function was also stronger in this condition. Kim et al. designed a brain microvascular model based on collagen I using 3D printing technology, as shown in Figure 3B [75]. They verified the effectiveness of the system by optical monitoring of fluorescently labeled molecules and barrier disruption by mannitol. In 2017, Wang et al. fabricated a BBB chip for in vitro drug permeability study [40]. The chip also incorporated electrodes to measure TEER, but the brain microvascular endothelial cells (BMECs) in the chip were induced by pluripotent stem cells (hiPSCs). Gravity-driven circulatory cavity filling proved that an effective BBB model can be achieved without high fluid shear stress. In addition, they tested the permeability of FITC-glucan, caffeine, cimetidine, and doxorubicin in the model, and the results were very similar to those in vivo.

The cells in the BBB system mainly include brain microvascular endothelial cells, astrocytes, and pericytes. The interactions between these cells can largely affect the function of the BBB [76]. The BBB chips mentioned above mostly focused on the study of brain microvascular endothelial cells without taking into account the interaction between cells. Jeong et al. designed a BBB-on-a-chip that co-cultured endothelial cells and primary astrocytes [77]. A 4 × 4 cross microchannel array on a single chip formed 16 BBB sites, which has the potential to meet medium-throughput drug screening. Wevers et al. designed an in vitro model of BBB based on a high-throughput plate [41]. Unlike the previous structure, the chip contained no artificial membrane. One side of the culture channel was filled with TY10 cells to form microvessels, and the other side was filled with astrocytes (hAst cells) and pericytes (hBPCT cells) to create BBB co-cultures. The chip will provide a new platform for studying large molecule drugs through BBB.

Except for a few small molecules that can cross the BBB through lipid-mediated free diffusion, most drugs need to be engineered to cross the barrier and be absorbed by the brain [78]. Papademetriou et al. used BBB chips to verify the effect of flow environment on binding and BBB penetration of AngiopEP-2-functionalized nanoparticles [42]. They found that the binding of Angiopep-2 coupled liposomes (Ang2-liposomes) to brain endothelial cells was reduced at relatively high fluid shear stress (6 dyne/cm^2^). In addition, the presence of fluid shear stress also enhanced the permeability of Ang2-liposomes in the BBB model. The cells cultured in the above study were immortalized brain endothelial cells, lacking astrocytes and pericytes, which were not complete for the construction of a physiological structure. The enhanced BBB model designed by Park et al. provided a good platform for solving this problem [79]. They exposed BBB chips co-cultured with three cell types to development-stimulated hypoxic differentiation and cultured them under physiological currents to obtain a BBB model that more closely approximates the in vivo physiological environment.

### 2.5. Heart-on-a-Chip

**Figure 4:**
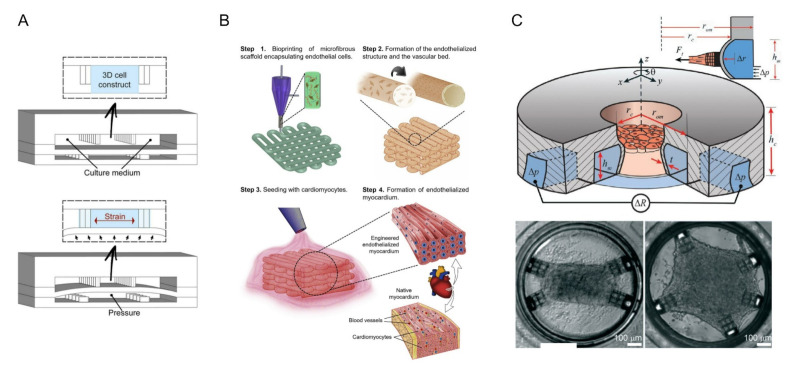
Heart-on-a-chip system. (**A**) Neonatal rat and human induced pluripotent stem cell-derived cardiomyocytes (hiPSC-CM) were suspended in a fibrin gel matrix and filled in the central channel. The PDMS membrane is deformed by pressurizing the bottom compartment. Due to the presence of openings between the columns, compression translates into the significant uniaxial strains applied to the 3D cell structure. Reprinted with permission from Ref. [43]. Copyright 2016 Royal Society of Chemistry. (**B**) Schematic diagram of 3D bioprinting manufacturing endothelialized myocardium. Reprinted with permission from Ref. [44]. Copyright 2016 Elsevier. (**C**) The seeding well in the middle was surrounded by a ring of micro-channels for driving and detection. The illustration shows a cross-section of the protruding wall. The “cage” on the wall of the device limited collagen remodeling and acted as an attachment point for cells. By customizing the position of the cage, different tissue shapes could be defined (rectangle and pentagon were shown in the picture) Reprinted with permission from Ref. [46]. Copyright 2021 Royal Society of Chemistry.

The heart is one of the least regenerative organs in the body but also one of the most important [80]. In recent years, cardiovascular health problems have been growing and have gained increased attention [81]. Therefore, it is very important to understand the pathogenesis of cardiovascular disease and prevent it. The advent of microfluidic chips has made it possible to study heart tissue in vitro. The beat of cardiomyocytes (CMs) is commonly used to assess the pumping and drug effects of the heart [82]. In 2011, Grosberg et al. designed a classic heart-chip structure using muscle membrane (MTF) technology [83]. They implanted neonatal rat CMs onto the elastic membrane to prepare the muscle membranes. When pacing with the electrodes, the CM contractions caused the muscle membrane to curl. The contractility of myocardial cells can be analyzed by measuring the degree of curl. On this basis, Agarwal et al. obtained chips with sub-millimeter MTF using engraving laser machines [84]. In addition, microfluidics devices had been conjoined with optical cardiac contractility measurements, which can be adapted for high-throughput cardiac tests. Marsano et al. considered the physiological microenvironment of cardiac myocytes and incorporated physiological mechanics into chip design, as shown in Figure 4A [43]. The experimental results showed that cyclic stress significantly increased the bonding complex, leading to the excellent physiological performance of the microengineered heart tissue.

In recent years, 3D printing technology and direct laser writing (DLW) photolithography have been increasingly used in the microfluidic field. Based on previous work [85], Zhang et al. fabricated endothelialized myocardial tissue using 3D bioprinting technology [44]. After the microfiber scaffold is printed, human umbilical vein endothelial cells (HUVECs) migrate around the fibers to form a vascular bed. Finally, cardiomyocytes were inoculated into the void of the scaffold to form an endothelialized myocardium (this process was performed on the hydrophobic PDMS surface) (Figure 4B). This can be used for drug screening when it is cultured in a microfluidic perfusion bioreactor. Alan et al. have recently shown that alginate and cross-linking agent precursor solutions have deleterious effects on cardiomyocytes [45]. The bioink for bioprinting contains alginate, which has been gelated, but it remains to be seen whether it will affect cardiomyocytes when tested in microfluidic bioreactors. Sun et al. developed a color hydrogel by polymerizing non-close-packed colloidal arrays onto super aligned carbon nanotube sheets (SACNTs) [86]. The anisotropy and electrical conductivity of the structure contribute to the arrangement and beating of cardiomyocytes. In addition, visual heart sensing can be achieved by combining color hydrogel with the microfluidic device where the cell pulsation causes optical changes in hydrogel deformation. Jayne et al. designed a heart chip with integrated microfluidic actuators and mechanical sensors [46]. The structure was fabricated by DLW and soft lithography, as shown in Figure 4C. Its innovation lies in 3D self-assembly and the growth of custom-shaped heart tissue and real-time monitoring of heart tissue stress. In addition, the relatively small size of the chip enables it to be integrated with a standard 384-well plate or standard 24-well plate, providing the possibility of high throughput screening.

### 2.6. Skin-on-a-Chip

When the skin is in contact with the external environment, it will be affected by ultraviolet rays, pollutants, and microorganisms in the environment. A variety of factors will lead to skin diseases [87]. Drug delivery through the skin has also been a research hotspot in recent years, including the detection of health markers by using microneedle patch skin tattoo [88] and the detection of drugs in vitro by using skin-on-a-chip platforms. To enable further industrial research on the skin penetration of drugs, harmful chemicals, and cosmetics, a platform to mimic the skin and its equivalents in a straightforward and simple manner is required. Therefore, a miniaturized chip based on microfluidics becomes a better choice [89].

Sorafenib [90] is a typical therapeutic agent for hepatocellular carcinoma, but there are many side effects in its clinical application, which includes skin reactions on the areas of the hands and feet. An on-chip skin driven by a gravity fluid system can be used to study this process [47,48]. The dermis layer cultured in the chip was composed of collagen-fibroblast, which was inoculated with keratinocytes to form the epidermis layer on dermis gel cultured for four days. The adverse reactions caused by sorafenib concentration and the exposure time in air were studied. Hemolysin and eosin (H&E) staining, immunohistochemistry staining, and real-time polymerase chain reaction (PCR) were used for characterization. The results showed that the thickness of the spinous layer and basal layer increased significantly, similar to the histological results obtained from patients with skin reactions on the hands and feet. Kim et al. [48] used a similar structure and characterization methods to perform drug tests on the natural product cosmetic ingredient *Curcuma longa* leaf extract (CLLE), which indicated that 50 μg/mL of CLLE showed the best enhancement of skin barrier function and anti-aging effect. The skin-on-a-chip integrated with a de-bubble setup and TEER electrode can be used to test the stimulation of 10 known toxins and non-toxins, and the results meet the standard of OECD 439 [49]. These results show that skin-on-a-chip can reproduce the structure and function of the epidermis well, showing great potential for irritation detection, for example in the pharmaceutical and cosmetic industries. By simplifying the operation process and designing high throughput, it will have more applications in the field of in vitro safety evaluation.

### 2.7. Other Single Organs-on-a-Chip

The vasculature of the human body is an important system involved in systemic circulation. It plays an indispensable role in human development, but it is also closely associated with many tumors and vascular diseases [91,92]. Asaumi et al. fabricated a vessel-on-a-chip model using laser and soft lithography [93]. By fluorescent labeling of tumor cells, they investigated the effects of chemokines, inhibitors, and vascular endothelial cells on tumor cell extravasation. The tumor cell migration was also demonstrated in the presence of a gradient in fetal bovine serum (FBS) concentration. Llenas et al. designed a vessel chip with serpentine microchannels to characterize infantile vascular tumors [50]. They took cells from human umbilical vein endothelial cells and coated them on the lining of the microchannels to replicate the cellular contents of natural human blood vessels. Fluorescent beads were added to enhance the accuracy of fluid measurements. In addition, they used clot-activated fibronectin coating and artificially increased channel permeability to simulate physiological thrombosis and bleeding, respectively. Salipante et al. also constructed a 3D microvascular platform to probe the mechanical properties of blood vessels [94]. These models provide a good platform for studying the pathogenesis of vascular diseases.

Compared with the traditional manufacturing process, 3D bioprinting technology can simulate the tissue structure more accurately. Abudupataer et al. co-cultured primary human aortic endothelial cells (HAECs) and human aortic smooth muscle cells (HASMCs) into microfluidic chips made of PMMA using 3D bio-printing technology [51]. A continuous media flow was infused to simulate blood flow in blood vessels. Zhou et al. also used 3D printing technology to make a vascular chip model with curved and straight channels [52]. The channel shape was found to affect the directional arrangement of endothelial cell morphology. They performed fusion and collapse tests on several pluronic-nanoclay inks and found that the P30-N12 ink had higher printability and fidelity. Unlike the 3D printing mentioned above, the 3D structures printed in this scheme are used as templates to cast PDMS to obtain chips with microchannels. The simplicity and flexibility of this method means it has great potential in preparing complex channel networks.

**Figure 5:**
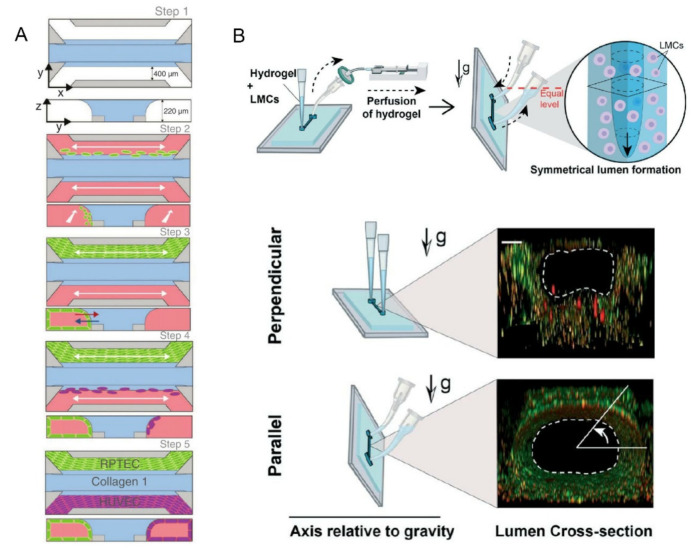
(**A**) The blue area was a liquefied ECM gel composed of type 1 collagen. The green and purple areas were tubules formed by human RPTEC and HUVEC. Reprinted with permission from Ref. [54]. Copyright 2022 American Society of Nephrology. (**B**) GLP technique to fabricate the lymphangion-chip with the figure below showed a fluorescence view of the cross-section of the lumen. Reprinted with permission from Ref. [55]. Copyright 2021 Royal Society of Chemistry.

The kidney plays a role in filtering and eliminating waste in the blood [95]. Various kidney-on-a-chip models have been used to study drug toxicity and related disease mechanisms [96]. While majority of the previously described models were dynamic cell cultures in chips, Tian et al. developed a liver-kidney-on-a-chip based on precision-cut tissue slices (PTSs) to investigate the liver and kidney tropism of extracellular vesicles (EVs) in breast cancer in vitro [53]. The results showed that breast cancer EV had stronger liver tropism and was more active than Transwells. They also demonstrated that chemokine (CXCL12) and its receptor (CXCR4) were involved in the liver tropism. Acute kidney injury (AKI) is usually associated with loss of proximal renal tubule function and cellular damage. To explore the effects of renal ischemia/reperfusion injury (rIRI) on proximal renal tubules and cells, Vormann et al. combined the OrganoPlate 3-lane with a standard 384-well plate to design a kidney-on-a-chip for high-throughput tests [54]. As shown in Figure 5A, the chip is made using a process similar to the BBB-on-a-chip [41] described in the previous section. They simulated rIRI by freely combining normoxic/low oxygen, with/without perfusion, and with/without glucose. The experimental results showed that the renal tubules were damaged by ischemia while the endothelium was not affected as adenosine had a protective effect on rIRI. The advantage of this platform is that it can well simulate the condition of rIRI and meet the requirements of high throughput, which is conducive to the research and treatment of AKI.

In addition to the previously mentioned on-chip models of common organs, Selahi et al. recently developed the first lymphangion-chip [55]. Based on the Saffman–Taylor instability principle, they constructed a 3D cylindrical lymphangion-on-a-chip with the gravitational lumen patterning (GLP) method (Figure 5B). The co-culture of lymphatic endothelial cells (LECs) and lymphatic muscle cells (LMCs) under specific medium perfusion and fluid shearing was achieved, and the cavity diameter and muscle layer thickness could be adjusted. This model can be applied well to the mechanism analysis of related diseases, the identification of new drug targets, and the testing of the potential therapeutic drugs.

### 2.8. Multi-Organ-on-Chip Systems

The multi-organ-on-chip systems have been developed to study the physiological interactions between multiple organs and even the drug responses of the whole human body. Using programmable liquid operation systems for controlling the valve pressure, various types of fluids could be added, mixed, and transported inside the robotic pharmaceutical pipelines [63,97,98,99,100]. Hence, multiple culture chambers, micro-wells, and bioreactors could be coupled together.

Many microfluidic components could be added to better control the fluidic flow, enhance real-time monitoring, and mimic physiological microenvironment in vivo. For example, to mimic the mixing of blood of central circulation system in vivo, an arteriovenous (AV) mixing reservoir is proposed to be integrated with the latter cell culture bioreactors [101]. Different from measuring drug or metabolite levels in outflows of individual organ chips, the AV could mix the fluidic samplings and enable them to more analogous to sampling peripheral blood in a patient. Multiple studies proposed methods for connecting multiple organs-on-a-chip systematically. A central microfluidic breadboard could be fabricated to control the fluidic flow between multiple external chambers/sensing units [102]. By using pipelines to connect these soft lithography-fabricated units with the breadboard, the physical status of cell culture and drug-related biomarkers could be monitored in line inside two-organs-on-a-chip. Another method is to connect multiple micro-organs in the same plate with multiple bioreactors and micro-wells, each chamber or trans-well containing different cellular types representing different organs—for example, inter-connected chambers containing liver, skin, bone marrow, and tumor. The configuration of these devices may vary depending on the number of organs to be coupled [103]. In some versions of these multi organ systems, the medium flows directly from one parenchymal cell type to another without or with an intermediate porous membrane [103,104,105]. For example, the fluid flows from hepatocytes in chamber one to skin cells in chamber two [106]. Recently, in vitro multi-organs-on-chip system perfuse the medium through the channels arranged by an endothelium. The endothelial cells are cultured to interface across porous membranes with organotypic tissues in adjacent channels or chambers so as to better replicate the vascular perfusion and the cross-endothelial transport of drugs and metabolites in the human body [101,104,105,107].

## 3. Applications

### 3.1. Pharmacokinetic (PK) and Pharmacodynamic (PD) Analysis

Drug development is a long process, and preclinical modeling and simulation can predict key PK and PD parameters well, thereby reducing the generation of toxic metabolites and drug side effects [107]. PK is mainly used to describe the body’s effects on drug effects, including drug absorption, distribution, metabolism, and excretion (ADME) in the body. PD is used to describe the effects of drugs on the body, that is, the changes of efficacy with time and concentration. There is a close relationship between them. Physiologically based PK (PBPK) models are increasingly being used in drug development and regulation, and have shown a promising ability to predict the quantitative extent of PK-based drug–drug interactions and the effect of age, genetics, disease, and formulation [108]. However, it still has limitations, such as an inability to take into account differences in organ-specific drug clearance, distribution, and absorption caused by differences in cellular uptake, transport, and metabolism [109]. Organ chips can restore part of organ function at a physiological level, providing a new solution to overcome these limitations.

At present, organ-on-chip models used to study drug metabolism are mainly divided into single-organ and multi-organ models. The single-organ model is used to study the metabolic process and the toxicity of drugs in designated organs. The multi-organ model is used to analyze the drug reaction process under the interaction of multiple organs in human body, including the concentration of a drug in multiple organs and the influence of metabolites on other organs. Most of the organ-on-chips mentioned earlier can be used as single-organ models for the study of drug metabolism. Here, a model not mentioned before is selected for brief introduction. Chi et al. developed a three-layer L-TumorChip system to study the pharmacokinetics of a drug (doxorubicin) for breast cancer [110]. The upper channel is used to culture the confluence layer of microvascular endothelial cells (HMVEC). The middle is a porous PDMS membrane, and a cylindrical chamber at the bottom contains cancer cells coated with matrix glue (MDA-MB-231). They measured the pharmacokinetics of cancer cells in the presence of HMVEC treated with or without doxorubicin by monitoring the fluorescence intensity induced by intracellular caspase-3 activity. The advantage of the chip is that it replicates the cancer-matrix interaction and can be extended to meet the requirements of high-throughput drug screening.

**Figure 6:**
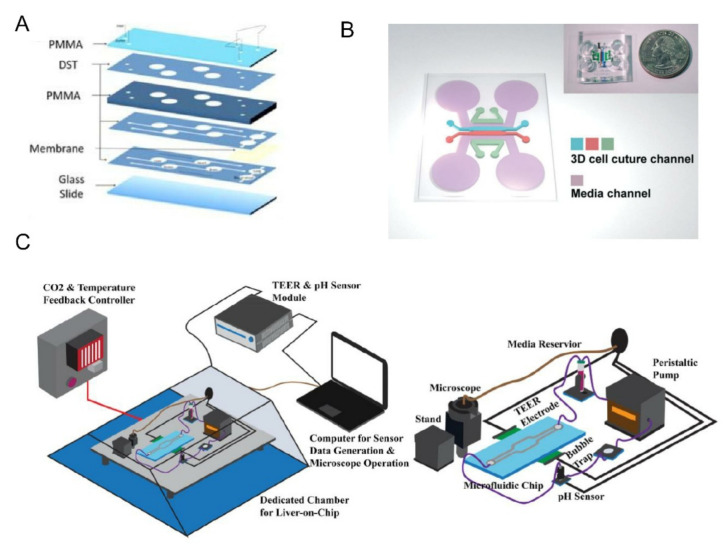
(**A**) Microfluidic devices were fabricated in a PDMS-free approach by layering PMMA and adhesive films with chambers, channels, and ports formed by laser cutting. Organoids were incorporated by being immobilized in hydrogels within each chamber. Semi-porous membranes enabled inclusion of planar vascular and lung modules. Reprinted with permission from Ref. [113]. Copyright 2020 IOP Publishing. (**B**) Schematic overview and photograph of the device to mimic natural sprouting morphogenesis from the pre-formed microvascular network. Reprinted with permission from Ref. [119]. Copyright 2016 Royal Society of Chemistry. (**C**) Schematic showing the organ-on-a-chip sensing system that could continuously measure electrical impedance and PH. The glass chip made it easy for the microscope to examine the cell monolayer in vivo. The cell index (CI) converted from the TEER sensor could be used as an indicator to evaluate the acute cytotoxicity of new drugs Reprinted with permission from Ref. [125]. Copyright 2020 IOP Publishing.

The multi-organ model has been a hot topic in recent years. Liu et al. first studied the pharmacokinetics of ginsenoside compound K (CK) using a multi-organ chip platform [111]. The chip adopts a multi-layer structure. COMSOL software is used to simulate the flow rate distribution in the system. Layers one to five are used to evaluate intestinal absorption, intravascular transport, liver metabolism (hepatotoxicity), renal excretion, and renal toxicity, respectively. The absorption and metabolism of CK were analyzed by LC-MS/MS. In addition, they also tested the cytotoxicity of CK in single organ chips, a liver-kidney combined chip and a multi-organ chip, and the results showed that CK metabolites produced by liver cells were more toxic to kidney cells than CK itself. In the multi-organ chip, CK was absorbed by the intestinal lumen, and almost no CK reached the liver and kidney cell layers through the vascular lumen. The results of controlled experiments show that it is of great significance to construct a multi-organ model to study drug metabolism. Shinha et al. used a PK-PD model based on a multi-organ chip to evaluate the inhibitory effects of simvastatin and ritonavir on the metabolism of an anticancer prodrug (CPT-11) [112]. The liver part is involved in metabolism and the cancer part is used as the drug target. The specific parameters obtained from the experiment can be calculated by the PK-PD model to evaluate the interaction between drugs. Skardal et al. designed a multi-organ chip system that integrates the liver, heart, lung, blood vessels, testis, colon, and brain, as shown in Figure 6A [113]. To avoid absorbing protein and drug compounds on the device wall, they chose PMMA as the chip material [114]. They used the platform to demonstrate that drugs recalled by the FDA can be toxic to humans, and that the platform can observe a more complex response than the toxicity detected by the single-organ chip.

The models mentioned above all integrate multiple organs into one chip. Herland et al. combined different double-layer organ chips and arteriovenous (AV) reservoirs with automatic pipetting processing instruments to form a first-pass multi-organ-chip system [101]. Prior to drug testing, drug flux between compartments, media flow, drug concentration, and drug absorption by the material need to be calculated. The vascular endothelial lining channel in the chip allows the whole system to use a common “blood substitute”, perfusion and fluid coupling. The advantage of the AV reservoir design is that the drug concentration in the vascular channels of all chips can be measured by sampling the AV reservoir (mass spectrometry analysis). The author’s team successfully used the system to predict the PK parameters for orally administered nicotine and observed the different toxicity of intravenously injected cisplatin to various organs. The system shows great potential in predicting drug PK parameters and toxicity, as well as the possibility of application in personalized medical and clinical trials in the future.

### 3.2. Nano-Medicine

Nano-medicine mainly involves drug delivery, drug therapy, in vivo imaging, in vitro diagnosis, biomaterials, and active implants [115]. Most applications in combination with microfluidic technology are toxicity testing of nanoparticles and drug delivery and treatment. While nanoparticles have shown great potential in nano-medicine, continued exposure to nano-materials could have adverse health effects. Lu and Chen et al. reviewed the adverse effects of different nanoparticles on several key organs in the human body and summarized the organ-on-chip platforms used to study the toxicity of nanoparticles in recent years [116,117].

We selected several recent research results of nano-medicine related to tumors for a brief introduction. Davila et al. reconstructed tumor micro-vessels on a chip to evaluate the interaction of polystyrene nanoparticles with endothelial cells [118]. A semicircular ridge is formed by the thermal imprint of the intermediate polymer impression (IPS) on the master mold, and semicircular micro-channels are formed by casting PDMS. Human umbilical vein endothelial cells are inoculated with fibronectin-treated channel surfaces and then infused with a medium containing nanoparticles. The obvious accumulation and internalized distribution of nanoparticles in cytoplasm were observed by high-magnification fluorescence microscope. Cancer cells are known to be the main source of angiogenic factors, and nano-drugs based on small interfering RNA (siRNA) and a nano-carrier have great potential in anti-angiogenesis. As shown in Figure 6B, Lee et al. constructed a cancer angiogenic chip based on a previously developed microfluidic device to verify the anti-angiogenic effects of siVEGF/MSN and siVEGFR/MSN [119]. The VEGF is vascular endothelial growth factor, VEGFR is its receptor, and MSN is mesoporous silica nanoparticles. The results of 3D imaging demonstrated that siVEGFR/MSN successfully inhibited tumor growth and angiogenesis, and had the ability to induce the normalization of cancer vessels [120]. The platform will facilitate further research into antiangiogenic nano-medicine and RNA interference (RNAi) based nano-medicine. With the continuous development of artificial intelligence, the combination of nano-medicine with automation and AI will become the development trend in the future [121]. Fang et al. developed an organoid chip that can simulate the peristalsis of human colon tumor organoids [122]. Each micropore on the chip is used to grow organoids individually, and the micropore array contracts and dilates under pressure driven by the surrounding pressure channel to simulate the mechanical stimulation of the physiological environment. They used the chip to evaluate the efficiency of poly(N-(2-hydroxypropyl) methylacrylamide-co-methacrylic acid-block-poly (methyl methacrylate) (P(HPMA-co-MAA)-b-PMMA) micelles as nano-materials, and compared the results with traditional non-peristaltic chips. The results showed that the uptake, accumulation, and cell mortality of PHPMA in the organoid of the microarray were significantly reduced compared with the traditional chips. Peristalsis interfered with the endocytosis of the organoid, leading to a retarded cell response to the nanoparticle treatment. An organoid-on-a-chip has great advantages in organ complexity and microenvironment replication, which will be the main direction of the in vitro exploration of nano-drug screening.

### 3.3. Dynamic Monitoring for Disease Modeling

The combination of sensors and organ chips makes it possible to continuously monitor the dynamic changes of physiological parameters. Zhang et al. developed an organs-on-chips system that integrates physical and biochemical sensors with an automatic fluidic operation system [102]. The breadboard is composed of a microfluidic and a pneumatic valve controlling layer. The microfluidic channel is designed to be semi-cylindrical so that the pneumatic valve can be completely sealed when it starts. Optical pH, oxygen sensors, and temperature probes are used to monitor the microenvironment in organ chips. Electrochemical sensors are used for continuous in situ monitoring of organoid biomarkers and have the ability to regenerate when the captured antigens are saturated. The fabrication of the electrochemical biosensors and chip integration in the system are described in detail in [123]. They applied heart and liver chips to organ modules to verify drug responses to capecitabine and acetaminophen. The results were similar to those recorded. The toxicity of doxorubicin (DOX) was subsequently tested with the liver-cancer-heart chip. The results showed that the drug would cause significant death of liver cancer organs but also induced severe cardiotoxicity. The system has advantages in continuous monitoring and could reduce manual intervention with automated control during the monitoring process. However, the absorption of drugs by materials and the complexity of platform assembly need to be further optimized. For small molecules involving redox active co-factors, the structure of bio-based redox capacitor (BBRC) can also be used for biological detection [124]. The structure stores charge by forming functional films or hydrogels on the electrode surface and repeatedly exchanges electrons with soluble redox substances. After systematic collection and analysis, biological information can be inferred. In addition, embedded electrodes based on indium tin oxide (ITO) can be used for TEER measurements, avoiding environmental interference from traditional invasive electrodes (Figure 6C). The transparent glass chip and ITO electrode provide easy access to 3D printed portable digital microscopes for imaging living cells [125].

### 3.4. Safety Assessment/Toxicity Evaluation

Another important reason why the organ-on-a-chip is promising is that it will help the FDA and large pharmaceutical companies to make an accurate assessment of the safety of drugs in the early stage of drug testing, so as to decide whether to proceed to complex and demanding animal and human trials. The toxicity of drugs exposed to human tissues and unknown safety concerns are major reasons for the failure of potential drug candidates. Since the cells for constructing the organ-on-a-chip can be directly derived from humans, the species differences between human and animal can be effectively avoided, and the toxicity of drugs to target organs and even the human body can be accurately predicted. Through the organ-on-a-chip experiment, unsafe drugs can be screened out from the beginning, which is beneficial for effectively shortening the drug development cycle and reducing investment losses caused by drug safety issues.

Drug toxicity is closely related to liver metabolism. Emulate has developed a type of liver chip with a multi-layered liver cell structure [126]. Primary hepatocytes are located in the upper parenchymal cell channel sandwiched by ECM, and liver non-parenchymal cells are located in the submembrane vascular channels, including liver sinusoidal endothelial cells, Kupffer cells, and astrocytes. The chip uses microfluidic technology to ensure that all cells are always at an ideal concentration level of the drug. The researchers used the chip to study the effects of drugs, such as TAK-85, on liver health, mitochondrial function, and reactive oxygen species. With the fabricated chip, the researchers confirmed the mechanisms of several drugs and compounds known to be hepatotoxic. It was found that one compound induced fibrosis in rat liver chips but had no effect on hepatocytes in human liver chips. When evaluating the adverse side effects of drug therapy, target organs and other organs where adverse effects are latent and may also be included, such as liver (hepatotoxicity), kidneys (nephrotoxicity), heart (cardiotoxicity), or brain (neurotoxicity). Multi-organ-on-chip systems have practical value for toxicity assessments, and can reduce the use of laboratory animals and improve the efficiency of drug evaluation in preclinical studies. Bovard et al. designed a lung/liver-on-a-chip for the toxicological assessment of inhaled compounds associated with the development of chronic obstructive pulmonary disease, asthma, and lung cancer [127]. Combining normal human bronchial epithelial (NHBE) cells cultured at the air—liquid interface (ALI) and HepaRG™ liver spheroids, the co-cultured tissues maintained metabolic activity for 28 days. Exposure of ALI cultures to various aerosols or aerosol mixtures, using AFB1 as an example toxicant, found that AFB1 was less toxic in the presence of HepaRG™ spheroid cultures.

## 4. Conclusions

In recent years, OoCs have attracted great attention and made great scientific development from single-organ chips, to multi-organ chips, to a human-on-a-chip. Compared to traditional in vitro techniques, OoCs provide many advantages in various important respects. However, with the increasing complexity of functions, higher requirements are placed on integrated system components. At present, OoCs cannot replace animal experiment, which has its own bottleneck in terms of chip technology and biological mechanisms. The first shortcoming of OoC detection technology is that most of the current detection methods are direct observation or measurement of functional parameters. However, systems should produce specific physiological responses to different biological stimuli. Therefore, it is necessary to continue to develop detection techniques for better biochemical analysis in microfluidic devices. In addition, the complexity of chip preparation also limits its development and hence the simple fabrication process for massive production of OoCs is desired. The second key question is the source of cells. At present, most immortal cell lines used are derived from tumor cells, which have the problem of tissue dysfunction. Primary human cells are a good choice, but there are limitations on donor resources and cost. The use of human iPS-induced cells may compensate for this deficiency. It is believed that these technical problems will be solved one by one in the future, and OoCs will be applied to more emerging fields.

In this paper, we summarize the typical structures and recent research achievements of several organs-on-a-chip, including lung, liver, intestine, BBB, heart, vascular, kidney, and tumor. The innovations in models applied to the fields of PK/PD, nano-medicine, continuous dynamic monitoring in disease modeling, and safety assessment are also discussed here. We believe OoC systems will become an important tool in these fields and in personalized medicine in the future.

## Figures and Tables

**Table 1:** Human disease and clinical responses replicated in single organ chips.

Human Organ Chip	Materials	Cell Types	Technology for Fabrications	Disease Model or Clinical Mimicry	Ref
**Lung**	PDMS	Epi and endo	Soft lithography	Nanoparticle transport and toxicity	[8]
	PDMS, collagen, fibrin gel	Endo (HUVEC), fibroblast (HLF and iPF-HLF), epi (SAEC)	Polyester track etch, soft lithography	Fibrotic αSMA-positive disease	[30]
	PDMS 3D Matrigel	Epi (HPAEpiC), endo (HUVEC)	Soft lithography	Nanoparticle toxicity	[31]
	Soft collagen–elastin gel, thin gold mesh, PDMS, polycarbonate	Epi (HAEpC), endo (VeraVec)	Collagen molecules self-assembled, soft lithography	The air–blood barrier and alveoli network	[32]
	Plastic	Epi	Laser-cut, polyester track etch	Viral infectivity, inflammation	[33]
**Liver**	PDMS	Hep	Soft lithography	Drug toxicity	[34]
	PDMS, PE membrane	Hep, endo, hepatic stellate, Kupffer	Soft lithography	Liver-specific functions	[35]
	PDMS, ECM gel	Hep, endo, hepatic stellate, Kupffer	Soft lithography	Drug efficacy	[36]
**Gut**	PDMS	Epi (Caco-2)	Soft lithography	Intestinal barrier function	[22]
	PDMS, PET membrane	Epi (Caco-2)	Soft lithography, photolithography	Differentiation, drug absorption, and metabolism	[37]
	Polycarbonate, PDMS, titanium, gold, PET membrane	Epi (hAEC)	Laser-cut, soft lithography	Real-time measurements of barrier function	[38]
	PDMS, PMMA frame	Epi (Caco-2), endo (HUVEC)	Soft lithography	Injury of intestine villus and inflammatory reactions	[39]
**BBB**	PDMS, polycarbonate membrane, Pt	Endo (HCMEC)	Soft lithography	Effect of inflammation cytokine	[26]
	PDMS, Ag/AgCl, polycarbonate membrane, silicone gasket	Endo (iPS)	3D print	Drug permeability	[40]
	Glass and polymers (OrganoPlate), ECM gel	Endo, pericyte, astrocyte	-	High-throughput drug screening	[41]
	PDMS, polycarbonate membrane	Endo (bEnd.3)	Soft lithography	Angiopep-2 coupled liposome transport	[42]
**Heart**	PDMS	Cardiomyocyte/iPS	Soft lithography	Drug concentration-response	[43]
	Bioink, PMMA, PDMS	Endo(HUVEC), cardiomyocyte	Bioprinting, soft lithography	Drug screening	[44]
	Super aligned carbon nanotube sheets, hydrogel, conductive methacrylated gelatin, PDMS	Cardiomyocyte	Polymerization, ultraviolet (UV) irradiation, soft lithography	Dynamic cardiomyocyte sensing and drug screening	[45]
	PDMS	Cardiomyocyte (iPS)	Direct laser writing (DLW) lithography and soft lithography	Response of cardiac under mechanical loading and pacing.	[46]
**Skin**	PDMS	Fibroblast, keratinocyte	Soft lithography	Skin side effects of sorafenib	[47]
	PDMS, polyester membrane	Fibroblast, keratinocyte	Soft lithography	Drug testing	[48]
	PMMA, PET membrane	Keratinocyte	Micromilling, track etch	Skin irritation and drug toxicity	[49]
**Vessel**	PDMS	Endo (HUVEC)	Soft lithography	Thrombi and hemorrhage	[50]
	Hydrogel, PMMA	Endo (HAEC), aortic smooth muscle	Bioprinting, numerical control engraving	Physiologic and pathologic process in vascular wall	[51]
	Pluronic, nanoclay, PDMS	Endo (HUVEC)	3D printed, soft lithography	Endothelial cell morphology	[52]
**Kidney**	PDMS, polycarbonate membrane	Liver and kidney precision-cut tissue slices, Endo (HUVEC), breast cancer	Soft lithography	Extracellular vesicles organotropism	[53]
	Glass and polymers (OrganoPlate), ECM gel	Epi (RPTEC), Endo (HUVEC)	-	Renal ischemia and reperfusion injury	[54]
**Lymphangion**	PDMS	Endo (LEC), muscle (LMC)	Soft lithography	Lymphatic inflammation	[55]

## Data Availability

Data is contained within the article.
